# Highly Efficient Quantum Sieving in Porous Graphene-like Carbon Nitride for Light Isotopes Separation

**DOI:** 10.1038/srep19952

**Published:** 2016-01-27

**Authors:** Yuanyuan Qu, Feng Li, Hongcai Zhou, Mingwen Zhao

**Affiliations:** 1 School of Physics, Shandong University, Jinan 250100, Shandong, China; 2 School of Physics and Technology, University of Jinan, Jinan 250022, Shandong, China

## Abstract

Light isotopes separation, such as ^3^He/^4^He, H_2_/D_2_, H_2_/T_2,_ *etc*., is crucial for various advanced technologies including isotope labeling, nuclear weapons, cryogenics and power generation. However, their nearly identical chemical properties made the separation challenging. The low productivity of the present isotopes separation approaches hinders the relevant applications. An efficient membrane with high performance for isotopes separation is quite appealing. Based on first-principles calculations, we theoretically demonstrated that highly efficient light isotopes separation, such as ^3^He/^4^He, can be reached in a porous graphene-like carbon nitride material via quantum sieving effect. Under moderate tensile strain, the quantum sieving of the carbon nitride membrane can be effectively tuned in a continuous way, leading to a temperature window with high ^3^He/^4^He selectivity and permeance acceptable for efficient isotopes harvest in industrial application. This mechanism also holds for separation of other light isotopes, such as H_2_/D_2_, H_2_/T_2_. Such tunable quantum sieving opens a promising avenue for light isotopes separation for industrial application.

Light isotopes such as hydrogen and helium have been widely used in both scientific research and industrial fields. The skyrocketing demand of ^3^He in cryogenic industries^1^, neutron detection and medical lung imaging^2^,^3^, and the wide usage of D_2_ and T_2_ in nuclear technologies have made isotopes separation a pressing need. Isotopes separation is crucial and inevitable for these applications. The isotopes of an element are separable only by mass-dependent processes, e.g. thermal diffusion and quantum effect, because they have nearly identical chemical properties. Recently, membrane-based approaches with low energy consumption and easy operation have been proposed for isotopes separation. Theoretical work revealed that several membranes including porous graphene and graphene allotropes with appropriate pores may be implementable for separating ^3^He from ^4^He through quantum tunneling effect^4^,^5^,^6^,^7^,^8^. However, none of them is able to meet the acceptable permeance with high selectivity for industrial application^9^. These theoretically-designed porous structures also face the synthetic difficulties in creating the desired pores of uniform size into graphene sheets.

In recent years, two-dimensional (2D) carbon nitride materials, such as graphitic carbon nitride (g-C_3_N_4_)^10^, have attracted considerable attention due to their various potential applications in gas separation^11^,^12^, solar energy conversion^13^,^14^,^15^, spintronic devices^16^,^17^ and energy storage^18^ *etc*. These membranes composed of C and N atoms with intrinsic regular and uniformly distributed subnanometer pores are ideal for gas separation with designable pore sizes. Lately, a new member of carbon nitride family, the C_2_N-*h*2D membrane, have been successfully synthesized via a simple bottom-up wet-chemical reaction^19^. This membrane with uniformly distributed hexagonal pores (as shown in Fig. 1(a)), was proposed for promising application in electronics, sensors, catalysis, as well as gas separation^11^,^19^,^20^.

In this paper, based on first-principles calculations, we theoretically present that under moderate tensile strain, highly efficient quantum sieving for light isotopes separation can be achieved in a porous graphene-like carbon nitride membrane (C_2_N-*h*2D) that has been synthesized in recent experiments. Both the selectivity and permeance for helium isotopes separation meet the requirement for industrial application, which is attributed to the tunable helium-membrane interaction under tensile strain. Additionally, this approach also holds for the separation of other light isotopes (e.g. H_2_/D_2_, H_2_/T_2_). The excellent mechanical property and tunable quantum sieving effect in this carbon nitride membrane opens a promising avenue for light isotopes harvest, as well as for wide range of energy or environmental applications.

## Results and Discussion

### Energy profiles

**Figure 1:**
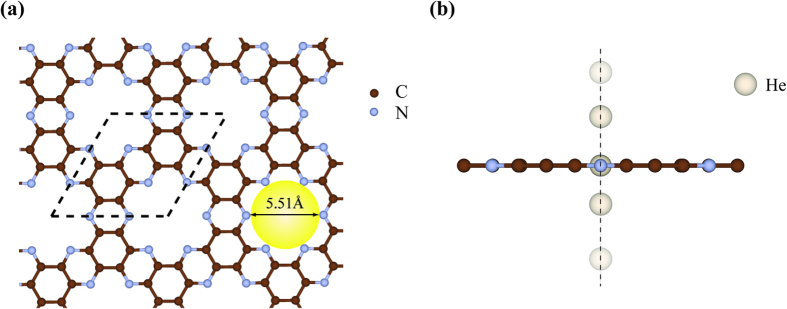
(**a**) Top view of C_2_N-*h*2D lattice. The brown and blue balls represent the C and N atoms respectively. The unit cell is indicated by the dashed rhombus and the inscribed circle is indicated by the yellow circle. (**b**) Side view of the penetration path of He passing through the pore of C_2_N-*h*2D. The white ball represents the He atom.

The optimized lattice constant for the unstrained C_2_N-*h*2D lattice is calculated to be 8.329 Å, in good agreement with experimental value^19^ (8.3 Å) and other theoretical values^11^,^20^ (8.354 Å or 8.32 Å). Figure 1(a) presents a top view of a fully relaxed C_2_N-*h*2D lattice, where the unit cell is indicated by a dashed rhombus. The pore size is characterized by the diameter of the inscribed circle, 5.51 Å, which is larger than the porous graphene or g-C_3_N_4_ membrane proposed for ^3^He separation^7^,^12^. As helium is a neutral atom without polarization, the interaction between the helium atom and the membrane is dominated by the weak vdW interaction. The lowest-energy approach to penetrate a symmetric pore is then simplified to be a straight line right through the center of the pore and perpendicular to the membrane, as shown in Fig. 1(b). Therefore, the energy profile for a helium atom passing through the pore of C_2_N-*h*2D membrane can be obtained by sequentially scanning the interaction energy between the helium atom and the membrane along the penetration path. The energy barrier along this path is mainly determined by the repulsive term of the vdW interaction, which is related to the overlap of the electron wavefunctions between the two close-shell systems due to the Pauli exclusion principle. Along this path, the maximum repulsive interaction between the helium atom and the membrane can therefore be tuned by modifying the pore size of the membrane, e.g. through applying tensile strain.

**Figure 2:**
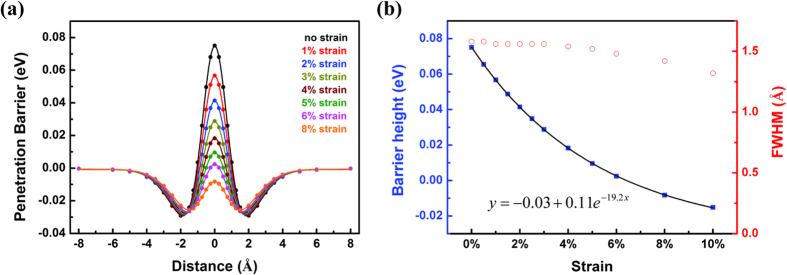
(**a**) Energy profile for He passing through the pore of the C_2_N-*h*2D membrane under different tensile strains. Colored points indicate the results obtained by first-principles calculations; while the curves show the numerically interpolated potentials. (**b**) The strain dependency of the penetration barrier heights and the FWHM.

Although the vdW interaction between the helium atom and the C_2_N-*h*2D membrane has no isotope effect, isotope separation can still be achieved via quantum tunneling with appropriate potential profiles due to their different masses, as described in the following parts. The selectivity and the permeance are two key parameters for ^3^He/^4^He separation in industrial application, where the industrial-acceptable values are considered to be 6 for selectivity and 6.7 × 10^−8^ mol/s/cm^2^/bar for permeance, repectively^9^. To study the tensile-strain-dependent selectivity and permeance, a series of biaxial tensile strains along the primitive vectors of the unit cell were applied to the C_2_N-*h*2D membrane to enlarge the pore size for different penetration properties. The biaxial tensile strain applied to the lattice is defined as the ratio of the deformation Δ*a* to the initial lattice constant *a*_0_, *ε = *Δ*a/a*_*0*_. Therefore, the lattice constant of the C_2_N-*h*2D membrane under strain *ε* is *a = a*_*0*_(1* + ε)*. The penetration energy profiles under different tensile strains are plotted in Fig. 2(a). As can be seen, the penetration barrier decreases with the increase of the tensile strain, which is understandable since larger pores would reduce the repulsive interaction between the membrane and the helium atom. The barrier height exhibits an exponential decay with a decay constant of 19.2; while the full-width-at-half-maximum (FWHM) of the barrier slightly decreases with the increase of the strain as shown in Fig. 2(b).

### Transmission probability

**Figure 3:**
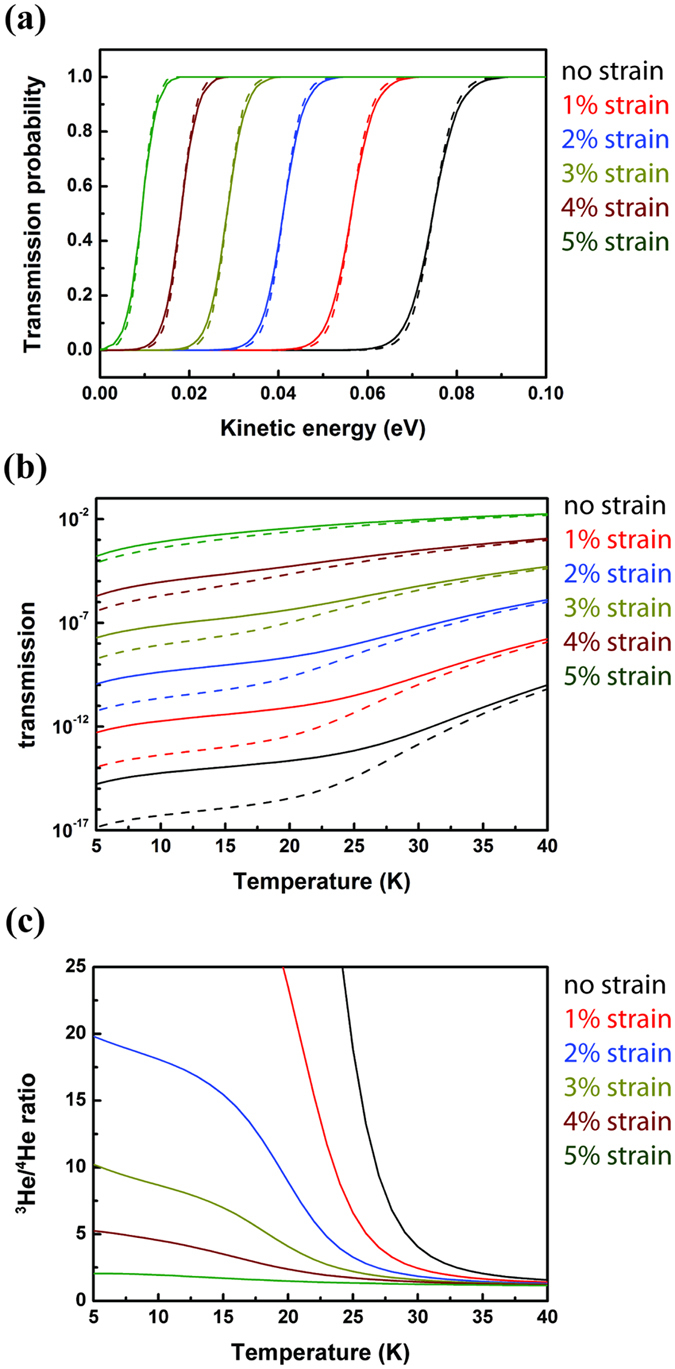
(**a**) Quantum-mechanical transmission probability of He passing through the pore of C_2_N-*h*2D membrane as a function of kinetic energy. The solid curves represent the quantum-mechanical transmission of ^3^He and the dashed curves represents that of ^4^He under different strains, respectively; (**b**) Thermally weighted transmission of ^3^He (solid curves) and ^4^He (dashed curves) under different strains; (**c**) ^3^He/^4^He transmission ratio under different strains.

Based on the energy profiles under different tensile strains, we performed one-dimensional (1D) finite difference calculations^21^ of the quantum tunneling probability *t(E)* as a function of kinetic energy *E* for each case, as shown in Fig. 3(a). In all cases, ^3^He transmission (solid curves) is preferred at low kinetic energy regimes while ^4^He transmission (dashed curves) becomes more likely at high kinetic energy regimes. Therefore, to enhance the selectivity of ^3^He from ^4^He, it is reasonable to keep the gas at a low temperature. It should be noted that although different exchange functional forms may give different kinetic crossovers of the transmission probability of ^3^He and ^4^He, the overall trend of the tunneling probability maintains^12^.

The thermally weighted transmission probability *p(T)* can be obtained via the same strategy utilized in our previous study^12^, 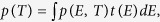 where 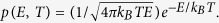 A classical Boltzmann distribution for the velocities of both ^3^He and ^4^He is assumed for simplification as suggested by previous studies^4^,^6^,^7^,^8^,^22^. The thermally weighted results under different strains at low temperature regime (5–40 K) are summarized in Fig. 3(b,c), including the thermally weighted transmission probability of ^3^He and ^4^He and the ^3^He/^4^He transmission ratio. As can be seen in Fig. 3(b), in all cases, the transmission probabilities of ^3^He deviate from ^4^He, due to their different masses impact on the quantum tunneling. The ^3^He/^4^He transmission decrease drastically as temperature increase under strains below 3% as shown in Fig. 3(c).

### Selectivity and permeance tuned by strain

**Figure 4:**
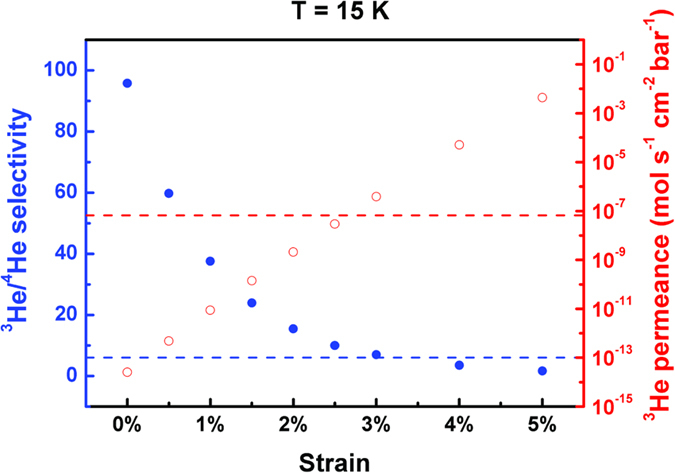
The ^3^He/^4^He selectivity and the ^3^He permeance under different strains at 15 K. The blue and the red dashed lines indicate the industrial acceptable values for the selectivity (6) and the permeance (6.7 × 10^−8^ mol/s/cm^2^/bar), respectively.

The total flux of He atom passing though the membrane can be estimated by: 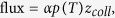 where *p(T)* is the thermally weighted transmission probability, 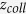 is the collision frequency between the particle and the pore, 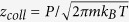 (*P* is the pressure, *T* is the temperature, *k*_*B*_ is the Boltzmann’s constant, *m* is the mass of the particle^4^), and *α* is the fraction of the area available for tunneling to occor: 
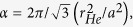
 where 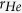 is the kinetic diameter of He atom and *a* is the lattice constant of the C_2_N-*h*2D membrane^23^. The permeance (*Q*) is defined as the ratio between the flux and the pressure drop across the pore, *Q* = flux/*P*, according to previous studies^8^,^9^. The selectivity and the permeance of ^3^He under different strains at 15 K are reported in Fig. 4, where the industrial acceptable values of the selectivity (>6) and the permeance (>6.7 × 10^−8^ mol/s/cm^2^/bar) are indicated by the blue and red dashed lines respectively^9^. It can be seen that at a specific temperature, the trade-off between the selectivity and the permeance can be adjusted by the strain. At 15 K, the acceptable selectivity and permeance are obtained at a strain of 3% with a selectivity of 7 and a permeance of 4 × 10^−7^ mol cm^−2^ s^−1^ bar^−1^ respectively. A more systematically scanning for the selectivities and the permeances at different temperatures can be found in Table 1, which showed that within the temperature range of 5 – 16 K, the industrial acceptable selectivity and permeance can always be satisfied by the proper adjustment of the tensile strain, where the most applicable strain for the C_2_N-*h*2D membrane is 3%, satisfying the industrial acceptable values from 5 to 16 K.

The dispersion interactions are critical for the potential energy barrier, we therefore calculated the penetration barriers based on the DFT-D3^24^ and DFT-D3(BJ)^25^ correction schemes (Supplementary Methods) for comparison (Supplementary Fig. S1 in SI). It is found that although different correction schemes did qualitatively change the potential energy barrier, the tensile strains that lead to the industrial acceptable selectivity and permeance are very close (Supplementary Table S1 and S2 in SI). The calculations based on the DFT-D3 scheme show that under a tensile strain of 2–3%, highly efficient ^3^He/^4^He separation at an industrial-acceptable level can be obtained at temperature ranging from 2 to 21 K; while for the DFT-D3(BJ) scheme, the most applicable strain becomes 3.5% with a temperature window of 5 to 16 K. Although the predicted strains and temperature windows slightly depend on the correction strategy of the dispersion interactions, the tunability of the penetration barrier and the selectivity by the tensile strain offer a promising approach for achieving highly efficient quantum sieving for light isotopes separation.

### Stability of the C_2_N-*h*2D membrane

**Figure 5:**
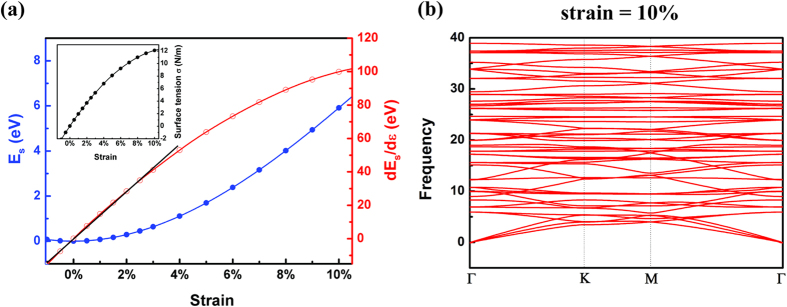
(**a**) Strain dependency of the strain energy *E*_*s*_ and its first derivative of the unit cell of the C_2_N-*h*2D lattice under different strains; the insert panel is the strain dependency of the surface tension σ. (**b**) Phonon spectra analysis of the C_2_N-*h*2D membrane under strain of 10%.

The stability of the C_2_N-*h*2D membrane under strain has also been examined by investigating the mechanical properties of the membrane, which can be revealed from the strain energy, *E*_*s*_ = *E*(*ε*) − *E*(0), where *E*(*ε*) represents the energy under strain *ε* and *E*(0) represents the energy under no strain. The strain energy *E*_*s*_ and its first derivative under different strains are plotted in Fig. 5(a). As can be seen, the strain energy increases as the increase of the tensile strain. The derivative d*E*_*s*_/d*ε* increases linearly with respect to the strain for strains below 3% (black line in Fig. 5(a)), corresponding to the harmonic region. This indicates that the C_2_N-*h*2D membrane under strain below 3% undergoes the elastic deformation, thus is quite stable. The surface tension σ increases to 12 N/m at a tensile strain of 10%, as shown in the insert panel of Fig. 5(a) (see the “Supplementary Equations” for the deduction of surface tension σ). To double check the stability of the C_2_N-*h*2D membrane, we also calculated the vibration frequencies of the phonon modes for a (3 × 3 × 1) supercell under strain of 10% as shown in Fig. 5(b), which indicated that even at tensile strain up to 10%, there is no imaginary frequency modes, implying the remarkable stability of C_2_N-*h*2D membrane under tensile strain.

Finally, we should emphasize that although we focus on helium isotopes separation in above discussion, the tunable quantum sieving mechanism also holds for other light isotopes separation, such as H_2_/D_2_, H_2_/T_2_. For instance, our calculations indicate that under tensile strain of about 3.5%, the selectivity and permeance for H_2_/D_2_ separation are able to meet the requirement for industrial application at low temperatures regime (below 30 K). Therefore, tensile strain can serve as a promising means to tune the efficiency of membranes for light isotopes separation and the porous graphene-like carbon nitrides would be ideal candidate materials to reach the goal.

## Methods

The first-principles calculations were performed within the density functional theory (DFT) using the plane-wave pseudopotential approach, implemented in the Vienna *Ab initio* Simulation Package (VASP)^26^,^27^,^28^. The electron-electron interactions are treated using a generalized gradient approximation (GGA) in the form of Perdew-Burke-Ernzerhof (PBE) for the exchange-correlation functional^29^. The van der Waals (vdW) interactions were included explicitly by using the empirical correction scheme of Grimme (DFT-D2)^30^. The energy cutoff of the plane waves was set to 520 eV with an energy precision of 10^−8^ eV. The atomic coordinates were fully relaxed using a conjugate gradient scheme without any symmetry restrictions until the maximum force on each ion was smaller than 0.001 eV/Å. Vacuum space larger than 15 Å was used to avoid the interaction between adjacent images. The Monkhorst-Pack meshes of 9 × 9 × 1 were used in sampling the Brillouin zone for the C_2_N-*h*2D lattice. In the subsequent calculations of the potential energy profiles for He atom penetrating the membrane pore, the z-coordinates of the atoms of C_2_N-*h*2D were kept fixed.

The quantum tunneling probability calculation was based on 1D finite difference calculations^21^, where the grid density was chosen to be 0.01 Å and a region of 1 Å located 7 Å away from the peak of the barrier was chosen for the incident planewaves as the barriers decrease below 0.001 eV at 6 Å away from the barrier peak.

## Additional Information

**How to cite this article**: Qu, Y. *et al.* Highly Efficient Quantum Sieving in Porous Graphene-like Carbon Nitride for Light Isotopes Separation. *Sci. Rep.* **6**, 19952; doi: 10.1038/srep19952 (2016).

## Supplementary Material

Supplementary Information

## Figures and Tables

**Table 1:** The selectivities (**S**) and the permeances (**Q**, mol/s/cm^2^/bar) under different strains for temperatures ranging from 4 to 17 K.

Temperature	4K		5K		8K		10K	
strain	S	Q	S	Q	S	Q	S	Q
2%	20.2	2.8 × 10^−10^	19.8	4.3 × 10^−10^	18.8	8.7 × 10^−10^	18.1	1.2 × 10^−9^
2.5%	14.2	3.8 × 10^−9^	13.6	5.9 × 10^−9^	12.4	1.2 × 10^−8^	11.8	1.6 × 10^−8^
3%	10.6	4.6 × 10^−8^	10.2	7.1 × 10^−8^	9.2	1.5 × 10^−7^	8.7	2.0 × 10^−7^
4%	5.4	4.3 × 10^−6^	5.3	7.1 × 10^−6^	4.9	1.7 × 10^−5^	4.5	2.4 × 10^−5^
**Temperature**	**12K**		**15K**		**16K**		**17K**	
**strain**	**S**	**Q**	**S**	**Q**	**S**	**Q**	**S**	**Q**
2%	17.3	1.5 × 10^−9^	15.5	2.0 × 10^−9^	14.5	2.3 × 10^−9^	13.4	2.6 × 10^−9^
2.5%	11.2	2.0 × 10^−8^	10.0	2.8 × 10^−8^	9.4	3.2 × 10^−8^	8.6	3.7 × 10^−8^
3%	8.1	2.5 × 10^−7^	7.0	3.7 × 10^−7^	6.5	4.2 × 10^−7^	5.9	4.8 × 10^−7^
4%	4.2	3.2 × 10^−5^	3.5	4.7 × 10^−5^	3.3	5.4 × 10^−5^	3.0	6.3 × 10^−5^

The red numbers identify the conditions satisfying the industrial acceptable selectivity (>6) and permeance (>6.7 × 10^−8^ mol/s/cm^2^/bar).
