# Supplementary material for: Highly Efficient Quantum Sieving in Porous Graphene-like Carbon Nitride for Light Isotopes Separation
Source: Sci Rep. 2016 Jan 27;6:19952. doi: 10.1038/srep19952 (PMC4728688; doi:10.1038/srep19952)
Supplement: Supplementary Information [file srep19952-s1.pdf]

Supplementary Information for

Highly Efficient Quantum Sieving in Porous Graphene-like Carbon Nitride for Light Isotopes Separation

Yuanyuan Qu[a], Feng Li[a,b], Hongcai Zhou[a], Mingwen Zhao[a]*

[a] *School of Physics, Shandong University, Jinan 250100, Shandong, China;*

[b] *School of Physics and Technology, University of Jinan, Jinan 250022, Shandong, China*

Supplementary Equations

Deduction of energy-temperature-dependent transmission probability p(E,T)

According to Maxwell-Boltzmann distribution, the distribution of the molecular velocities at temperature *T* in one-dimension is given by:

$$p(v,T)=\left(\frac{m}{2\pi k_B T}\right)^{1/2} e^{-\frac{mv^2}{2k_B T}}.$$

Therefore, the thermally weighted transmission can be obtained by

$$p(T)=\int p(v,T)t(\frac{1}{2}mv^2)dv=\int p(E,T)t(E)dE,$$

where *t(E)* is the quantum tunneling probability as a function of kinetic energy as introduced in the main text.

As such, we can obtain:

$$
\begin{aligned}
p(E,T) &= p(v,T)\frac{dv}{dE} \\
&= \left(\frac{m}{2\pi k_B T}\right)^{1/2} e^{-\frac{E}{k_B T}} \frac{1}{\sqrt{2Em}} \\
&= \left(\frac{1}{4\pi k_B T E}\right)^{1/2} e^{-\frac{E}{k_B T}}
\end{aligned}
$$

Deduction of surface tension σ

Similar to the force f in one-dimension and the pressure P in three-dimension, the surface tension σ in two-dimension can be obtained by the partial derivative of the surface energy E with respect to the surface area S:

$$\sigma = \frac{\partial E}{\partial S}.$$

For the unit cell of C_2N-*h*2D lattice at no strain, the surface area is $S_0 = \frac{\sqrt{3}}{2} a_0{}^2$, where a_0 is the lattice constant. For the lattice under strain ε, $S = \frac{\sqrt{3}}{2} a^2 = \frac{\sqrt{3}}{2} \left(a_0 (1+\varepsilon)\right)^2$.

Therefore, we can obtain the surface tension as a function of the tensile strain as follows:

$$
\begin{aligned}
\sigma &= \frac{\partial E}{\partial S} \\
&= \frac{\partial E}{\partial \varepsilon}\frac{\partial \varepsilon}{\partial S} \\
&= \frac{\partial E}{\partial \varepsilon}\frac{1}{\sqrt{3} a_0^2 (1+\varepsilon)}
\end{aligned}
$$

Supplementary Figure

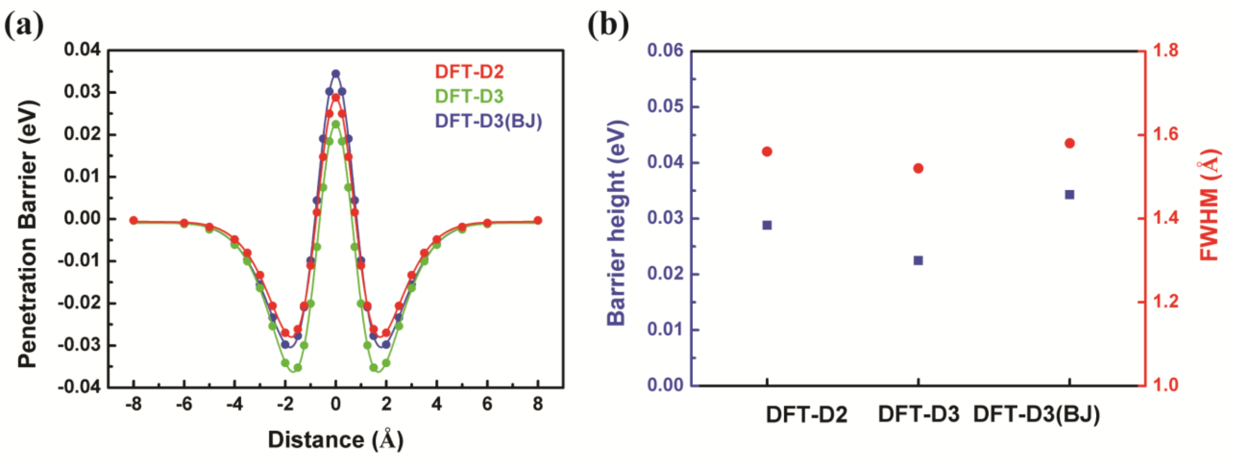

Figure S1 (a) Comparison between the energy profiles for He passing through the pore of the C_2N-*h*2D membrane by using DFT-D2, DFT-D3 and DFT-D3(BJ) correction schemes under the strain of 3%. Colored points indicate the results obtained by first-principles calculations; while the curves show the numerically interpolated potentials. (b) Comparison between the barrier height and the FWHM by using DFT-D2, DFT-D3 and DFT-D3(BJ) correction under the strain of 3%.

Supplementary Table

Table S1 The selectivities (**S**) and the permeances (**Q**, mol/s/cm^2/bar) under different strains for temperatures ranging from 1 to 22 K by using DFT-D3 correction. The red numbers identify the conditions satisfying the industrial acceptable selectivity (> 6) and permeance (> 6.7 $\times10^{-8}$ mol/s/cm^2/bar).

**Temperature**	**1K**		**2K**		**5K**		**10K**	
**strain**	**S**	**Q**	**S**	**Q**	**S**	**Q**	**S**	**Q**
1.5%	22.9	3.4×10^{-13}	22.9	5.7×10^{-11}	21.1	8.5×10^{-10}	18.4	2.0×10^{-9}
2%	18.5	6.8×10^{-12}	18.4	1.1×10^{-9}	15.7	1.7×10^{-8}	12.3	4.1×10^{-8}
2.5%	9.1	1.3×10^{-10}	9.0	2.1×10^{-8}	8.4	3.1×10^{-7}	7.4	7.4×10^{-7}
3%	8.6	1.6×10^{-9}	8.3	2.7×10^{-7}	7.0	4.2×10^{-6}	5.4	1.1×10^{-5}
3.5%	3.7	1.8×10^{-8}	3.7	3.0×10^{-6}	3.8	5.3×10^{-5}	3.4	1.7×10^{-4}
**Temperature**	**15K**		**18K**		**21K**		**22K**	
**strain**	**S**	**Q**	**S**	**Q**	**S**	**Q**	**S**	**Q**
1.5%	16.1	3.2×10^{-9}	14.0	4.2×10^{-9}	10.4	6.0×10^{-9}	9.0	9.4×10^{-9}
2%	10.3	6.5×10^{-8}	8.7	8.7×10^{-8}	6.5	1.4×10^{-7}	5.7	1.5×10^{-7}
2.5%	6.5	1.2×10^{-6}	5.5	1.6×10^{-6}	4.2	2.6×10^{-6}	3.8	2.7×10^{-6}
3%	4.4	1.9×10^{-5}	3.7	2.6×10^{-5}	2.9	4.1×10^{-5}	2.7	4.3×10^{-5}
3.5%	2.9	2.9×10^{-4}	2.5	4.0×10^{-4}	2.2	6.0×10^{-4}	2.1	6.1×10^{-4}

Table S2 The selectivities (**S**) and the permeances (**Q**, mol/s/cm^2/bar) under different strains for temperatures ranging from 4 to 17 K by using DFT-D3(BJ) correction. The red numbers identify the conditions satisfying the industrial acceptable selectivity (> 6) and permeance (> 6.7 ×10^{-8} mol/s/cm^2/bar).

**Temperature**	**4K**		**5K**		**8K**		**10K**	
**strain**	**S**	**Q**	**S**	**Q**	**S**	**Q**	**S**	**Q**
2.5%	19.5	2.1×10^{-10}	19.6	3.5×10^{-10}	19.4	8.4×10^{-10}	18.8	1.2×10^{-9}
3%	12.8	3.1×10^{-9}	12.9	5.2×10^{-9}	12.8	1.2×10^{-8}	12.3	1.7×10^{-8}
3.5%	7.8	4.9×10^{-8}	8.1	8.0×10^{-8}	8.3	1.9×10^{-7}	8.1	2.6×10^{-7}
4%	4.0	6.8×10^{-7}	4.2	1.1×10^{-6}	4.5	2.5×10^{-6}	4.6	3.6×10^{-6}
**Temperature**	**12K**		**15K**		**16K**		**17K**	
**strain**	**S**	**Q**	**S**	**Q**	**S**	**Q**	**S**	**Q**
2.5%	17.9	1.6×10^{-9}	15.9	2.3×10^{-9}	15.0	2.6×10^{-9}	13.8	2.9×10^{-9}
3%	11.7	2.2×10^{-8}	10.2	3.3×10^{-8}	9.5	3.8×10^{-8}	8.7	4.3×10^{-8}
3.5%	7.7	3.5×10^{-7}	6.7	5.2×10^{-7}	6.2	5.9×10^{-7}	5.7	6.9×10^{-7}
4%	4.5	4.8×10^{-6}	4.1	7.1×10^{-6}	3.9	8.2×10^{-6}	3.6	9.5×10^{-6}

Supplementary Methods

Calculation of DFT-D3 and DFT-D3(BJ) correction schemes

The DFT-D3 and DFT-D3(BJ) correction schemes for the potential barriers are calculated by the Grimme's DFTD3 program, which is a open-source program developed by Lori A. Burns. The program can be freely downloaded at http://www.psicode.org/psi4manual/master/dftd3.html.

Supplementary Data

The Optimized Coordinates of the unit cell of unstretched C_2N-h2D lattice

(Cartesian coordinates, in unit of Angstroms)

C 3.449333 1.143009 0.000000

C 2.714540 2.415711 0.000000

C 9.063802 3.558728 0.000000

C 7.594219 3.558730 0.000000

C	4.879661	1.143025	0.000000
C	5.614455	2.415723	0.000000
C	9.044185	6.070146	0.000000
C	9.778977	4.797444	0.000000
C	3.429715	3.654427	0.000000
C	4.899298	3.654425	0.000000
C	6.879063	4.797432	0.000000
C	7.613857	6.070131	0.000000
N	2.756981	0.000000	0.000000
N	1.378485	2.387614	0.000000
N	6.950515	2.387617	0.000000
N	5.572031	0.000000	0.000000
N	2.786019	4.825541	0.000000
N	5.543002	4.825539	0.000000

The Optimized Coordinates of unit cell of unstretched C_2N-h2D lattice with a He atom

(Cartesian coordinates, in unit of Angstroms)

C	3.449666	1.143360	0.000000
C	2.714949	2.415821	0.000000
C	9.063581	3.559180	0.000000
C	7.594247	3.559178	0.000000
C	4.879239	1.143380	0.000000
C	5.613857	2.415843	0.000000
C	9.043633	6.069697	0.000000
C	9.778350	4.797236	0.000000
C	3.429718	3.653877	0.000000
C	4.899052	3.653878	0.000000
C	6.879442	4.797214	0.000000
C	7.614059	6.069677	0.000000
N	2.758745	-0.000046	0.000000

N	9.708306	2.389084	0.000000
N	6.949546	2.389102	0.000000
N	5.570286	0.000046	0.000000
N	11.114025	4.823973	0.000000
N	5.543753	4.823956	0.000000
He	0.000000	0.000000	0.000000

Sample VASP input file

INCAR file for optimization:

```
SYSTEM =C2N-h2D
Start parameter for this run
  PREC = Accurate
  ISTART = 0
  ICHARG = 2
  ENCUT = 520
Electronic relaxation
  NELM = 80
  NELMIN = 2
  EDIFF = 1.E-08
  LREAL = Auto
  ALGO = Normal
  GGA = PE
Ionic relaxation
  EDIFFG = -0.001
  NSW = 500
  IBRION = 1
  ISIF = 2
  POTIM = 0.50
DOS related values
  EMIN = -10
```

EMAX = 10

NEDOS = 800

ISMEAR = 0

SIGMA = 0.05

Write flags

LWAVE = F

LCHARG = F

LORBIT = 12

LVDW = T

INCAR file for static energy calculation:

SYSTEM =C2N-h2D

Start parameter for this run

PREC = Accurate

ISTART = 0

ICHARG = 2

ENCUT = 520

INIWAV = 1

Electronic relaxation

NELM = 80

NELMIN = 2

NELMDL = -5

EDIFF = 1.E-08

LREAL = Auto

ALGO = Normal

GGA = PE

Ionic relaxation

EDIFFG = -0.001

NSW = 0

IBRION = -1

ISIF = 2

POTIM = 0.50

DOS related values

EMIN = -10

EMAX = 10

NEDOS = 800

ISMEAR = -5

SIGMA = 0.05

Write flags

LWAVE = F

LCHARG = F

LORBIT = 12

LVDW = T
